# Computation of High-Performance Concrete Compressive Strength Using Standalone and Ensembled Machine Learning Techniques

**DOI:** 10.3390/ma14227034

**Published:** 2021-11-19

**Authors:** Yue Xu, Waqas Ahmad, Ayaz Ahmad, Krzysztof Adam Ostrowski, Marta Dudek, Fahid Aslam, Panuwat Joyklad

**Affiliations:** 1 School of Civil Engineering, Southwest Jiaotong University, Chengdu 610031, China; 2 Department of Civil Engineering, COMSATS University Islamabad, Abbottabad 22060, Pakistan; ayazahmad@cuiatd.edu.pk; 3 Faculty of Civil Engineering, Cracow University of Technology, 24 Warszawska Street, 31-155 Cracow, Poland; krzysztof.ostroski.1@pk.edu.pl (K.A.O.); marta.dudek@pk.edu.pl (M.D.); 4 Department of Civil Engineering, College of Engineering in Al-Kharj, Prince Sattam Bin Abdulaziz University, Al-Kharj 11942, Saudi Arabia; f.aslam@psau.edu.sa; 5 Department of Civil and Environmental Engineering, Faculty of Engineering, Srinakharinwirot University, Nakhonnayok 26120, Thailand; panuwatj@g.swu.ac.th

**Keywords:** support vector regression, AdaBoost, random forest, machine learning, high-performance concrete

## Abstract

The current trend in modern research revolves around novel techniques that can predict the characteristics of materials without consuming time, effort, and experimental costs. The adaptation of machine learning techniques to compute the various properties of materials is gaining more attention. This study aims to use both standalone and ensemble machine learning techniques to forecast the 28-day compressive strength of high-performance concrete. One standalone technique (support vector regression (SVR)) and two ensemble techniques (AdaBoost and random forest) were applied for this purpose. To validate the performance of each technique, coefficient of determination (R^2^), statistical, and k-fold cross-validation checks were used. Additionally, the contribution of input parameters towards the prediction of results was determined by applying sensitivity analysis. It was proven that all the techniques employed showed improved performance in predicting the outcomes. The random forest model was the most accurate, with an R^2^ value of 0.93, compared to the support vector regression and AdaBoost models, with R^2^ values of 0.83 and 0.90, respectively. In addition, statistical and k-fold cross-validation checks validated the random forest model as the best performer based on lower error values. However, the prediction performance of the support vector regression and AdaBoost models was also within an acceptable range. This shows that novel machine learning techniques can be used to predict the mechanical properties of high-performance concrete.

## 1. Introduction

Concrete is the most commonly used material in construction [1,2,3,4,5]. One of the necessary components of concrete is its binder, i.e., cement. There are threats to the environment caused by the process of cement production, including its high energy demand and the emission of numerous gases [6,7,8]. In order to overcome these threats, one option is to utilize additional materials that have binding properties, such as supplementary cementitious materials (SCMs), including silica fume, fly ash, blast furnace slag (BFS), etc., in place of cement, either during cement production or while manufacturing concrete [9,10,11]. Utilizing SCMs in concrete provides numerous advantages, including greater ultimate strength, increased durability, economic benefits, the prevention of surface cracking, and increased sustainability [12]. SCMs can be used to make a variety of concretes, including low-carbon concrete (LCC), self-compacting concrete (SCC), high strength concrete (HSC), and high-performance concrete (HPC) [13,14,15]. The American Concrete Institute defines HPC as ‘concrete that meets unique combinations of performance and uniformity requirements that cannot always be met using standard constituents and traditional mixing, pouring, and curing techniques’ [16]. Although several definitions for HPC have been provided, it is generally understood that HPC is concrete that is more durable than conventional concretes (and this includes its increased compressive strength capacity) [17]. For many years, HPC has been employed in the construction of several concrete structures because of its superior features, which include high strength, durability, and efficiency [18,19]. These qualities are often obtained by incorporating SCMs (especially silica fume) into HPC. In other words, SCMs can be regarded as a necessary component of HPC. The utilization of SCMs in HPC results in reduced costs, reduced heat production, decreased porousness, and enhanced chemical resistance (due to high tightness caused by the usage of a new generation of admixtures in concrete mixtures), all of which contribute to the lower maintenance costs associated with structures created using HPC [20,21].

In recent years, machine learning (ML) algorithms have demonstrated significant potential for forecasting cementitious material properties [22,23,24,25,26,27,28]. Among the numerous machine learning methods, support vector regression (SVR) and artificial neural network (ANN) methods have been widely utilized to predict concrete parameters such as compressive strength (C-S) [29], split-tensile strength, elastic modulus, and so on [30,31,32]. ANN and SVR, however, are standalone models. Other fields of study have demonstrated that prediction accuracy can be greatly improved by integrating the results of standalone models into an ensemble machine learning (EML) model [30]. So far, there has been limited research in this sector that employs ensemble learning to predict concrete parameters. Adaptive Boosting (AdaBoost) and random Forest (RF) are ensemble learning techniques that can improve prediction accuracy by combining numerous regression tree predictions and voting on the final outcome [6,33]. Ahmad et al. [6] performed standalone and EML techniques to predict the C-S of concrete and compared their accuracy. It was determined that EML techniques predicted the outcomes with a higher level of accuracy than the standalone technique. However, the results of the standalone technique were also within an acceptable range. Song et al. [34] conducted an experimental study alongside the application of standalone techniques to forecast the C-S of concrete containing ceramic waste. It was concluded that the prediction model’s outcomes were in good agreement with the experimental results. Abuodeh et al. [35] used the ANN technique to forecast the C-S of ultra-HPC and reported that the ANN model performed effectively when predicting outcomes. Hence, the present research focuses on the use of advanced techniques to forecast the properties of concrete.

Substantial research is currently being carried out to determine the mechanical characteristics of HPC. However, the casting of specimens in the laboratory and the curing of them to the desired age, in addition to testing, are time-consuming activities that require great effort. The use of novel techniques such as ML to forecast the mechanical characteristics of HPC may overcome these issues and eliminate the cost of experimentation. This study adopted both standalone (SVM) and EML (AdaBoost and RF) techniques to foretell the 28-day C-S of HPC. The performance of each model was validated using the coefficient of determination (R^2^) value. Additionally, statistical error checks (including checks on the mean absolute error (MSE) and the root mean square error (RMSE)) and k-fold cross-validation checks were used to compare the performance of each technique employed. Sensitivity analysis was also performed to determine the contribution of input parameters towards the prediction of outcomes.

## 2. Data Description

**Figure 1:**
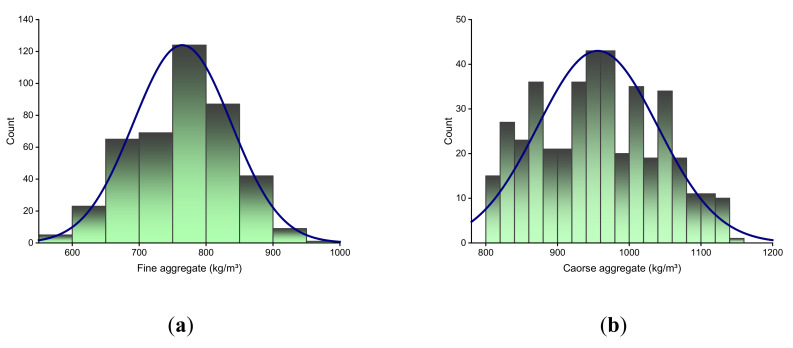

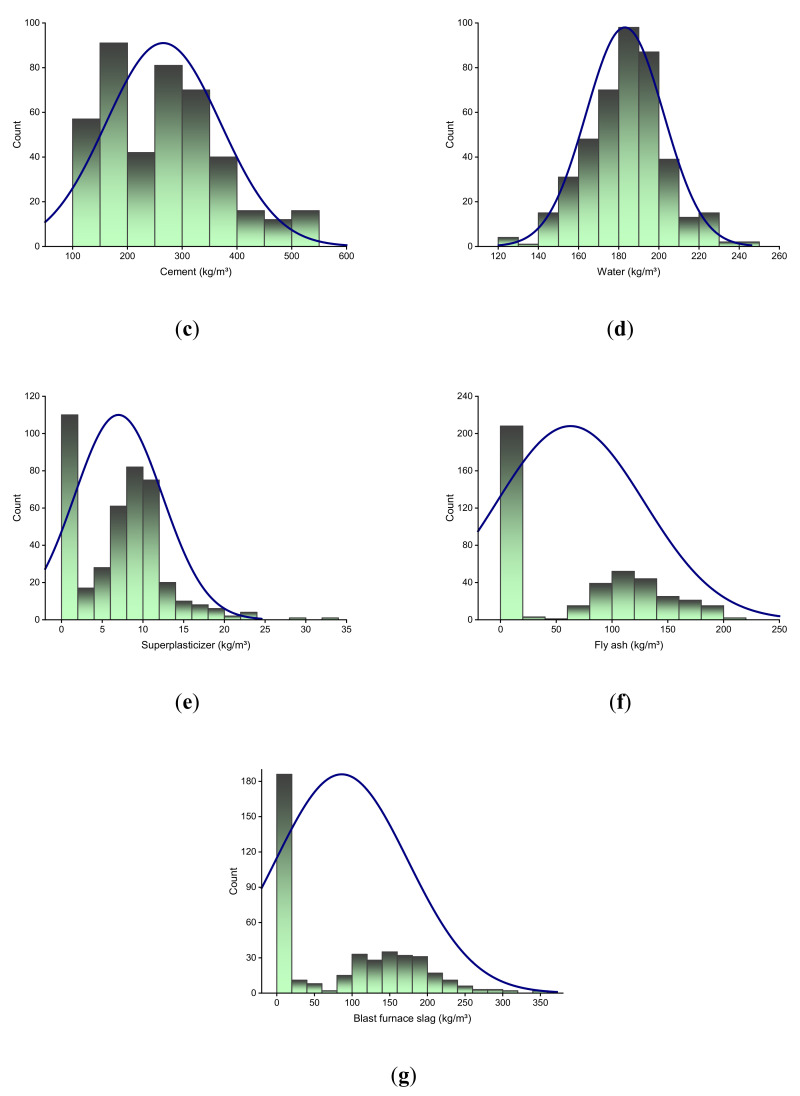
Relative frequency distribution of input variables: (**a**) fine aggregate; (**b**) coarse aggregate; (**c**) cement; (**d**) water; (**e**) superplasticizer; (**f**) fly ash; (**g**) blast furnace slag.

ML techniques require a variety of input variables to produce the expected output [36,37]. The data used to forecast the C-S of HPC were retrieved from the literature [38,39,40,41]. The total database available in the literature was 1030, but the data retrieved were filtered to retain only the 28-day C-S results for further studies. The models comprised fine aggregate, coarse aggregate, cement, water, superplasticizer, fly ash, and BFS as inputs, with only one variable, C-S, as the output. The quantity of data points and the input variables have a substantial impact on the model’s output [22,23,42]. In the case of the 28-day C-S prediction of HPC, this study employed a total of 425 data points (mix proportions). A descriptive statistical analysis for each input parameter was performed, and the results are shown in Table 1. This table gives the information of mean, median, mode, standard deviation, range, minimum, and maximum values for each input variable used in this study. In addition, the relative frequency distribution of each of the input variables used is shown in Figure 1.

## 3. Research Strategy

**Figure 2:**
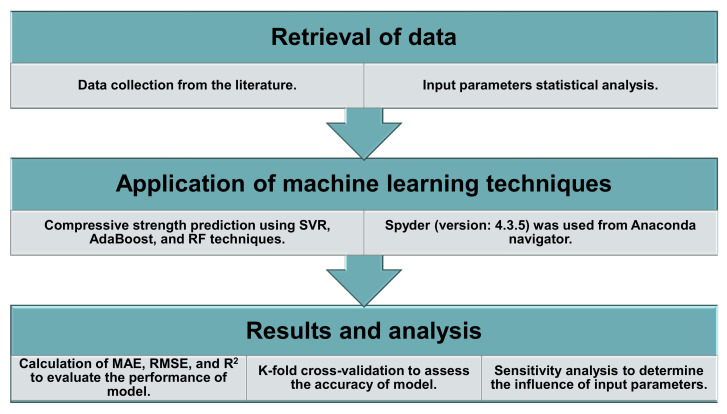
Sequence of research methodology.

Anaconda software [43] was used to run the ML models using Python code. The Anaconda navigator is a desktop graphical user interface included in the Anaconda software, which enables launching applications that guide Conda packages, environments, and channels without the need to utilize command-line methods. It is also a distribution point for Python and R programming languages used for data science and ML applications that focus on clarifying package building and maintenance. To estimate the C-S of HPC, this study applied three approaches: SVR, AdaBoost, and RF. Spyder (version: 4.3.5) was picked from the Anaconda navigator for executing the models. The R^2^ value of the expected outcome from all models indicated the degree of accuracy. R^2^ values normally vary between 0 and 1, with a bigger number indicating better precision between the actual and expected results. Furthermore, to evaluate the performance of all models used in this research, statistical checks, error evaluation (including MAE, RMSE), and k-fold cross-validation checks were performed. A sensitivity analysis was also conducted in order to determine the contribution of each input variable. This approach is depicted in Figure 2 as a flowchart.

### 3.1. Random Forest

The fandom forest technique has been extremely successful as a general-purpose classification and regression tool. The strategy, which mixes many randomized decision trees and averages their predictions, has demonstrated a superior performance in scenarios where the number of variables is significantly greater than the number of observations. Additionally, it is adaptable to both large-scale problems and to a variety of ad hoc learning challenges, returning measurements of varying relevance.

### 3.2. AdaBoost

Boosting is a machine learning technique based on the concept of constructing a highly accurate prediction rule by combining many very ineffective and erroneous rules. Freund and Schapire’s AdaBoost algorithm was the first practical boosting algorithm and continues to be one of the most widely used and studied algorithms, with applications in a wide variety of industries. The AdaBoost regressor is a supervised machine learning technique that works in an ensemble. It is also referred to as Adaptive Boosting since the weights are re-allocated to each instance, with greater weights being assigned to instances that were mistakenly classified. Most of the time, boosting methods are used for supervised learning to reduce bias and variation. These ensemble algorithms are used to make the weak learner stronger, and they are quite effective. They employ an n-fold increase in the number of decision trees during the training phase for the given data. As the initial decision tree/model is prepared, recorded data that have been improperly categorized are given a high priority. Only these data are transmitted as input to the next model. The procedure is repeated until a specified number of base learners has been generated. When it comes to binary classification tasks, the AdaBoost regressor is the most effective way to improve the performance of decision trees. It can also be used to improve the performance of any other machine learning algorithms that are currently in use.

### 3.3. Support Vector Machine

Support vector machines are a traditional machine learning technology that can still be used to address classification issues involving large amounts of data and are especially beneficial for multidomain applications running in a big data environment. However, support vector machines are theoretically complicated and computationally expensive. A support vector machine (SVM) is a machine learning technique that uses examples to learn how to label objects. For example, an SVM may be trained to spot fraudulent credit card activity by reviewing hundreds or thousands of data on both fraudulent and legitimate credit card activity. Alternatively, an SVM can be trained to recognize handwritten numerals by inspecting a vast collection of scanned images of handwritten zeros, ones, and so on. Additionally, SVMs have been effectively applied to an expanding number of biological applications. Automatic classification of microarray gene expression profiles is a common biomedical application of support vector machines.

## 4. Results

### 4.1. Statistical Analysis

**Figure 3:**
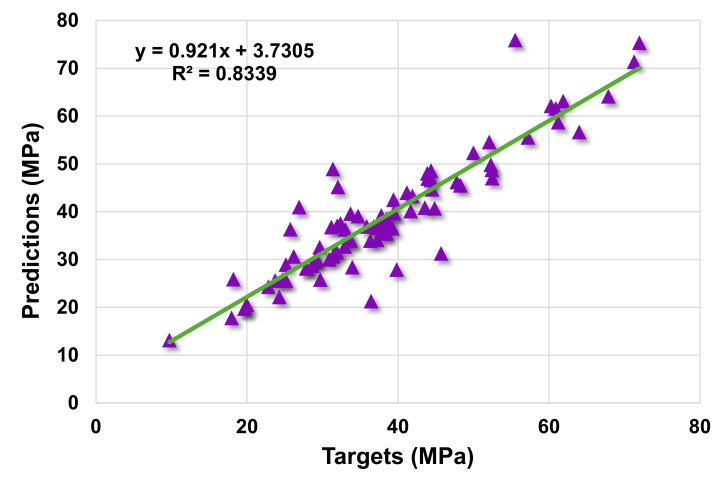
Actual and predicted outcomes relationship for support vector regression model.

**Figure 4:**
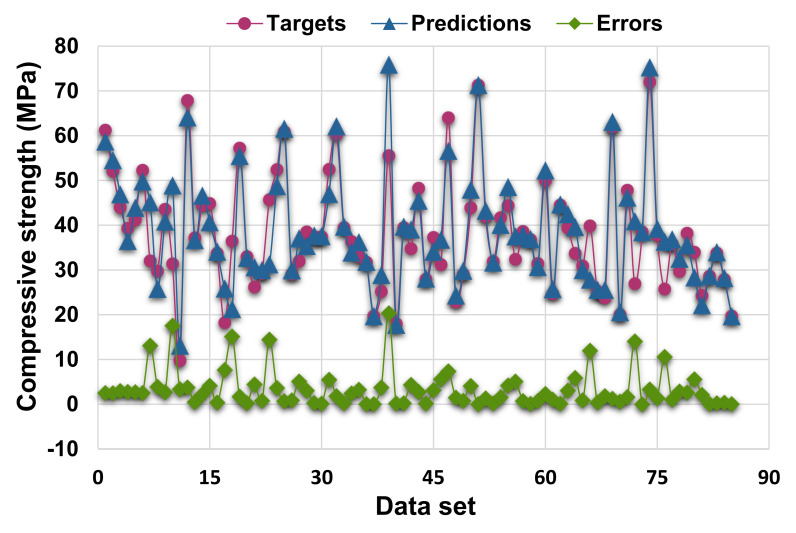
Actual, predicted, and error values’ distribution for support vector regression model.

Figure 3 demonstrates the statistical analysis interpretation of the real and anticipated results for the 28-day C-S of HPC using the SVR model. The SVR produced outcomes within an acceptable range and with a low divergence amongst the real and anticipated values. The R^2^ value of 0.83 reflects the model’s satisfactory performance in terms of predicting results. Figure 4 depicts the scattering of experimental values (targets), projected values, and errors for the SVR model. The distribution’s greatest, lowest, and average error values were 20.34, 0.01, and 3.33 MPa, respectively. It was observed that 34.1% of the error data were less than 1 MPa, 29.4% of the error data were between 1 and 3 MPa, 27.1% were between 3 and 10 MPa, and only 9.4% of the error data were larger than 10 MPa. These values indicate the good agreement between the predicted and actual results.

**Figure 5:**
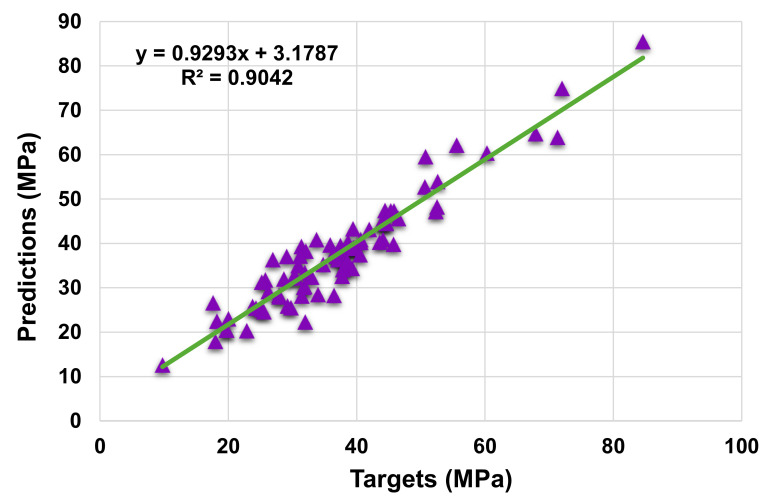
Actual and predicted outcomes relationship for AdaBoost model.

**Figure 6:**
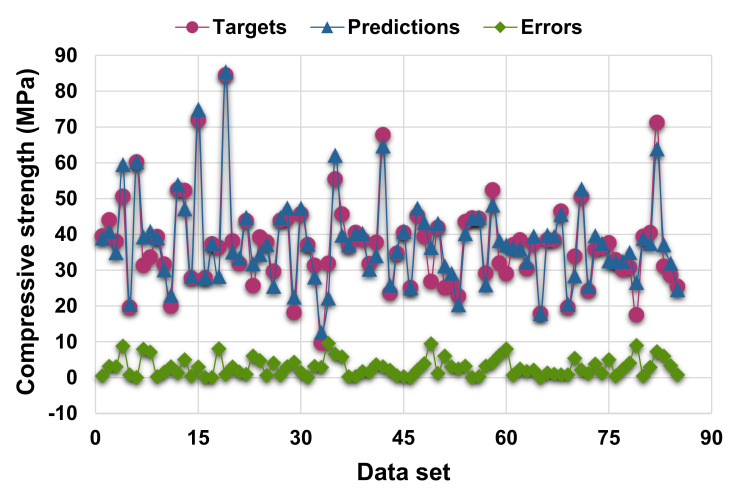
Actual, predicted, and error values’ distribution for AdaBoost model.

Figure 5 and Figure 6 illustrate the difference between the AdaBoost model’s actual and projected outcomes. Figure 5 illustrates the correlation between actual and projected results, with an R^2^ value of 0.90, which is higher than the SVR model, indicating the superior performance of the AdaBoost technique compared to the SVR. Figure 6 illustrates the distribution of actual values (targets), predicted values, and errors for the AdaBoost model. The maximum, minimum, and average error values of the distribution were 9.63, 0.01, and 2.95 MPa, respectively. It was found that 30.6% of error values were less than 1 MPa, 24.7% were between 1 and 3 MPa, 23.3% were between 3 and 5 MPa, and 21.2% were greater than 5 MPa. The R^2^ and error distribution of the SVM and AdaBoost models suggests that the AdaBoost model can predict the C-S of HPC more accurately.

**Figure 7:**
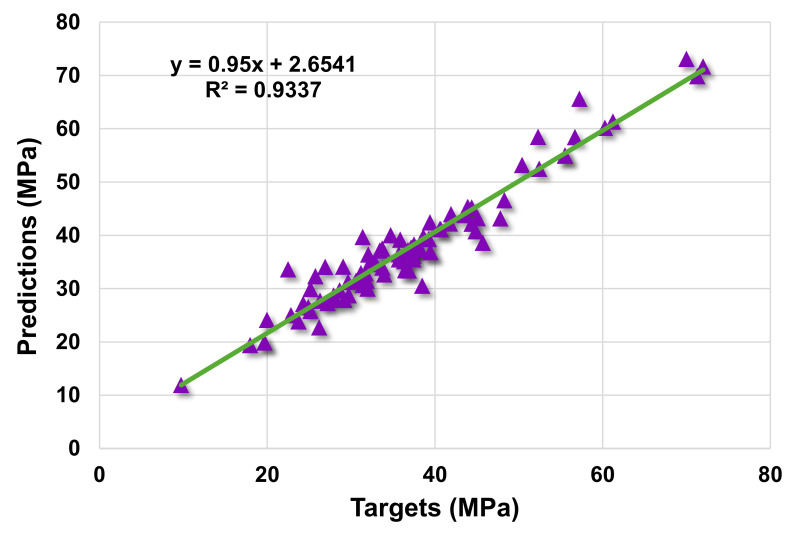
Actual and predicted outcomes relationship for random forest model.

**Figure 8:**
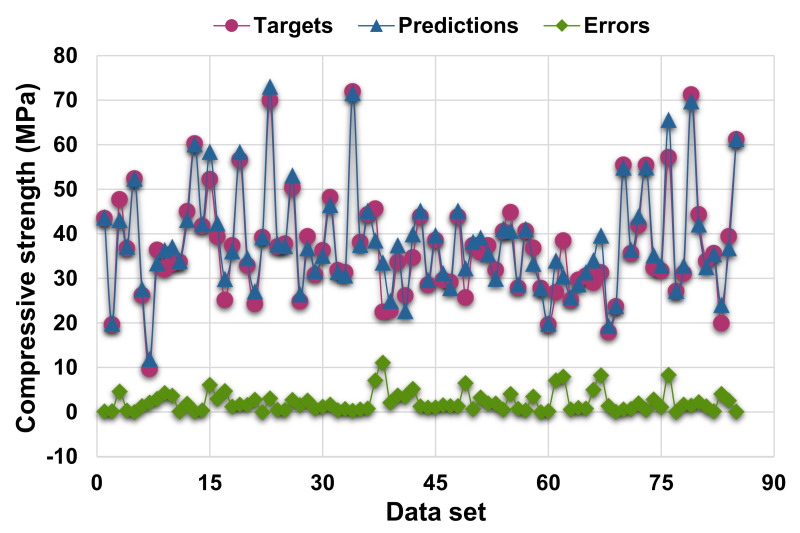
Actual, predicted, and error values’ distribution for random forest model.

Figure 7 shows the correlation between actual and projected results for the RF model. The R^2^ value for the RF model is 0.93, which demonstrates a higher level of accuracy than the SVM and AdaBoost models. Moreover, Figure 8 depicts the RF model’s distribution of actual values (targets), forecast values, and errors. The distribution’s maximum, minimum, and average error values were 11.09, 0.02, and 2.22 MPa, respectively. It was noted that 37.6% of error data were less than 1 MPa, 36.5%% were between 1 and 3 MPa, 14.1% were between 3 and 5 MPa, and only 11.8% were greater than 5 MPa. This analysis reveals that the RF model has a higher level of accuracy than the SVM and AdaBoost models due to greater R^2^ and lower error values. Furthermore, both EML algorithms (AdaBoost and RF) employed a total of twenty sub-models to discover the optimal value that yields an uncompromised output result. Hence, these results confirm that EML techniques can predict the outcomes with higher level of accuracy than the standalone techniques.

### 4.2. K-Fold Cross-Validation Checks

During execution, the model’s legitimacy was determined using the k-fold cross-validation approach. Generally, the k-fold cross-validation process is performed to determine the model’s validity [36], in which pertinent data are randomly dispersed and split into 10 groups. During this study, nine groups were used for training, while one was used to validate the model. In total, 80% of the data was used to train the models, while the remaining 20% was used to evaluate the employed models. The fewer errors made (MAE and RMSE), the larger the R^2^ value and the more accurate the model. Additionally, the process must be repeated ten times to reach a satisfactory result. This comprehensive approach results in an exceptional level of precision. Additionally, as illustrated in Table 2, statistical analysis of errors (MSE and RMSE) was undertaken for all models. These checks also supported the higher level of accuracy of the RF model due to lower error values when compared to the SVM and AdaBoost models. The model’s response to prediction was assessed using statistical analysis in accordance with Equations (1) and (2), which were acquired from the literature [44,45].

(1) $$\mathrm{MAE}=\frac{1}{n}\overset{n}{\underset{i=1}{\sum }}\left|x_{i}-x\right|$$

(2) $$\mathrm{RMSE}=\sqrt{\sum \frac{{\left(y_{pred}-y_{ref}\right)}^{2}}{n}}$$

where n = total number of data samples, x, $y_{ref}$ = reference values in the data sample, and $x_{i}$, $y_{pred}$ = predicted values from models.

**Figure 9:**
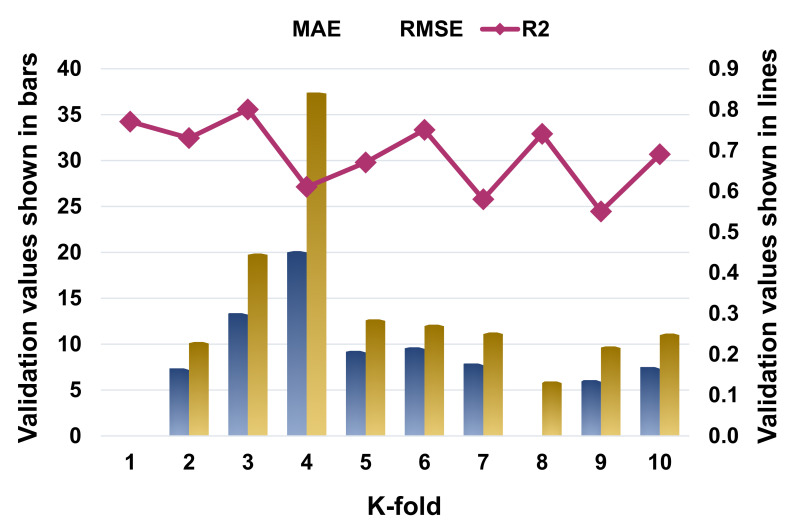
Statistical representation for K-fold cross-validation for support vector regression model.

**Figure 10:**
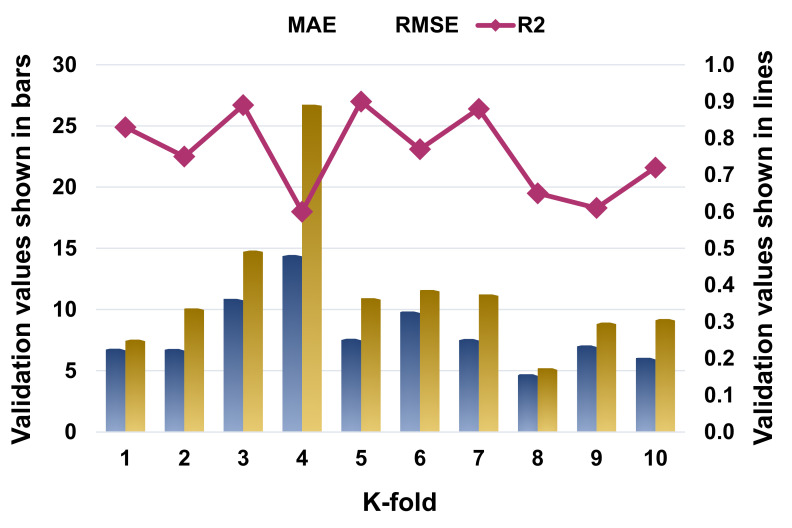
Statistical representation for K-fold cross-validation for the AdaBoost model.

**Figure 11:**
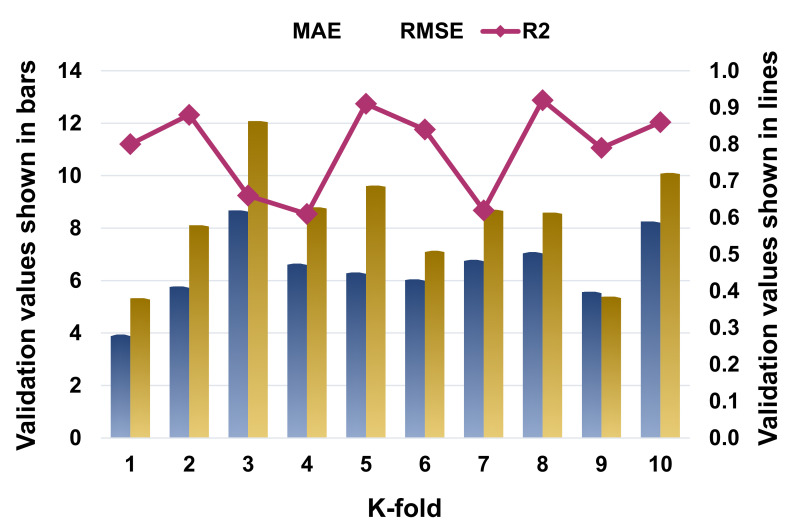
Statistical representation for K-fold cross-validation for random forest model.

The k-fold cross-validation was evaluated using R^2^, MAE, and RMSE, and their distributions for the SVR, AdaBoost, and RF models are presented in Figure 9, Figure 10 and Figure 11, respectively. The maximum, minimum, and average R^2^ values for the SVM model were 0.80, 0.55, and 0.69, respectively, as depicted in Figure 9. In comparison, the AdaBoost model’s greatest, lowest, and average R^2^ values were 0.90, 0.60, and 0.76, respectively (Figure 10). The RF model’s highest, lowest, and average R^2^ values were 0.92, 0.61, and 0.79, respectively (Figure 11). When the error values (MAE and RMSE) were compared, the average MAE and RMSE values for the SVM model were 9.04 and 13.62, respectively, whereas the average MAE and RMSE values for the AdaBoost model were 8.18 and 11.63, respectively, and the average MAE and RMSE values for the RF model were 6.51 and 8.39, respectively. The RF model with the lowest error and a high R^2^ value performed best when predicting results. The k-fold analysis results for all the employed models containing the values of MAE, RMSE, and R^2^ are listed in Table 3.

### 4.3. Sensitivity Analysis

**Figure 12:**
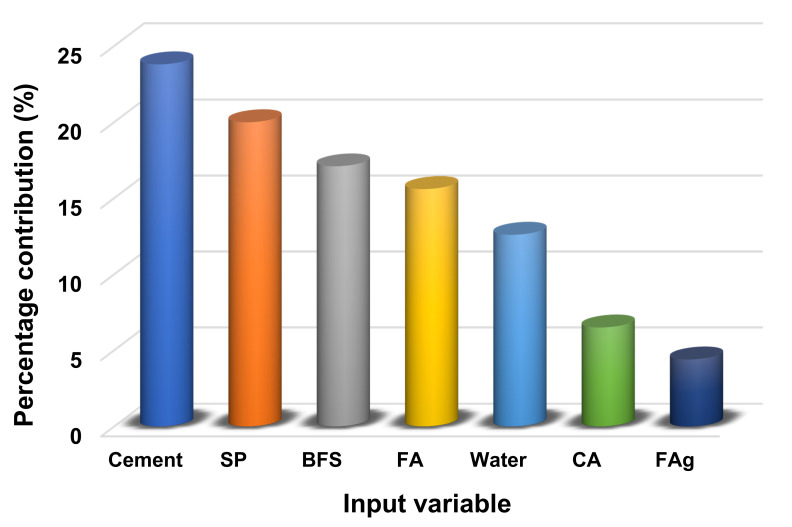
Contribution of input variable towards the prediction where SP: superplasticizer; BFS: blast furnace slag; FA: fly ash; CA: coarse aggregate; FAg: fine aggregate.

The purpose of this evaluation is to determine the impact of input variables on forecasting the C-S of HPC. The input parameters have a considerable influence on the projected outcome [24]. Figure 12 illustrates the effect of each input parameter on the C-S prediction of HPC. It was revealed from this analysis that cement was the most significant factor, with a 23.8% contribution, followed by superplasticizer, with 20.0%, and BFS, with a 17.1% contribution. However, the remaining input variables contributed to the prediction of C-S of HPC to a lesser degree, with fly ash accounting for 15.6%, water accounting for 12.6%, coarse aggregate accounting for 6.5%, and fine aggregate accounting for 4.4%. Sensitivity analysis yielded findings proportionate to the amount of input variables and number of data points used in the model’s construction. The results of the contribution level of all the input parameters can be obtained directly from the software used to run the models. However, Equations (3) and (4) were applied to ascertain the influence of each input variable on the model’s output:

(3) $$N_{i}=f_{max}\left(x_{i}\right)-f_{min}\left(x_{i}\right)$$

(4) $$S_{i}=\frac{N_{i}}{\sum_{j-i}^{n}N_{j}}$$

where $f_{max}\left(x_{i}\right)$ and $f_{min}\left(x_{i}\right)$ are the highest and lowest of the anticipated outputs over the $i^{th}$ output.

## 5. Discussion

**Figure 13:**
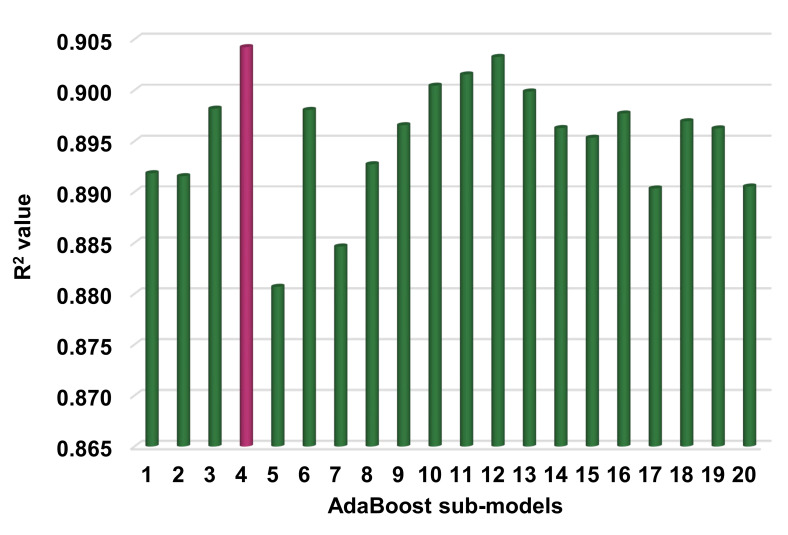
AdaBoost sub-model’s coefficient correlation (R^2^) values.

**Figure 14:**
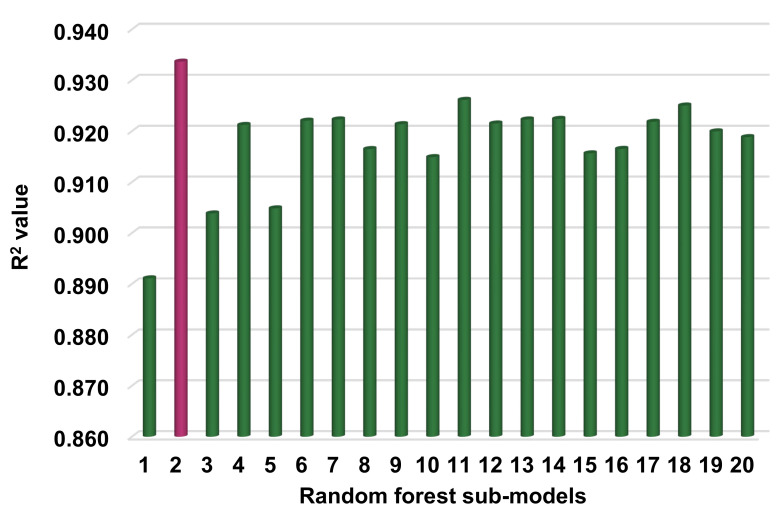
Random forest sub-model’s coefficient correlation (R^2^) values.

The goal of this research was to illustrate how supervised ML approaches could be applied to forecast the compressive strength of HPC. The study employed three machine learning techniques: one standalone, i.e., SVR, and two ensembled, including AdaBoost and RF. To ascertain which algorithm was the most accurate predictor, the prediction performance of each technique was compared. The result of the RF model was more precise, with an R^2^ value of 0.93, compared to the SVM and AdaBoost models, which produced R^2^ values of 0.83 and 0.90, respectively. Additionally, the performance of each model was confirmed using statistical analysis and the k-fold cross-validation technique. The lower the error levels, the better the model performed. However, assessing and proposing the optimal ML regressor for predicting results across a range of topics is difficult, as the success of each model was highly dependent on the input parameters and the data points used to run it. However, EML techniques often exploit the weak learner by building sub-models that can be trained on data and optimized to maximize the R^2^ value. Figure 13 and Figure 14 illustrate the distribution of R^2^ values for sub-models in the cases of the AdaBoost and RF approaches, respectively. The maximum, minimum, and average R^2^ values for AdaBoost sub-models were 0.904, 0.881, and 0.895, respectively (Figure 13), whereas the maximum, minimum, and average R^2^ values for RF sub-models were 0.934, 0.891, and 0.918, respectively (Figure 14). These values suggest the higher accuracy of RF sub-models when compared to AdaBoost sub-models. The literature indicates that RF models yield more accurate results than other ML approaches [46]. Additionally, a sensitivity analysis was conducted to ascertain the effect of each input parameter on the predicted C-S of HPC. The performance of the model can be affected by the input variables and the size of the data set. The sensitivity analysis examined the extent to which each of the seven input parameters contributed to the anticipated result. This study compared the performance of three ML approaches in order to determine the best technique for forecasting the C-S of HPC. The RF approach was noted to be the more accurate technique for the prediction of the mechanical properties of concrete. The contribution of this study is the selection of a superior/more accurate approach for predicting concrete strength.

## 6. Conclusions

This study aimed to employ standalone and ensemble machine learning (EML) techniques to predict the 28-day compressive strength (C-S) of high-performance concrete (HPC). One standalone technique, i.e., support vector regression (SVR), and two EML techniques, namely AdaBoost and random forest (RF), were employed to predict outcomes. The following conclusions have been drawn from this research:

EML techniques were more accurate in predicting the C-S of HPC than the standalone technique, with the RF model exhibiting the highest accuracy. The coefficient correlation (R^2^) values for the SVR, AdaBoost, and RF models were 0.83, 0.90, and 0.93, respectively. The results of all of the models employed were within an acceptable range, with little variance from the actual results.

Statistical analysis and k-fold cross-validation checks also demonstrated the models’ good performance. Additionally, these checks confirmed the RF model’s superior performance compared to the other models studied.

The contribution of input parameters was determined by sensitivity analysis and observed that cement, superplasticizer, blast furnace slag, fly ash, waster, coarse aggregate, and fine aggregate contributed towards the outcome’s prediction by 23.8%, 20.0%, 17.1%, 15.6%, 12.6%, 6.5%, and 4.4%, respectively.

Novel machine learning methods can accurately forecast the strength properties of concrete without the need for excessive sample casting and testing time.

This paper proposes the use of both standalone (SVM) and ensembled (AdaBoost and RF) machine learning approaches to forecast the 28-day C-S of HPC. Other ML techniques should also be employed to compare their accuracy in predicting outcomes. It is recommended that in future investigations, the quantity of data points and outcomes be increased through experimental work, field tests, and numerical analysis employing a variety of methodologies (e.g., the Monte Carlo simulation, among others). In order to improve the models’ responses, environmental variables (such as high temperatures and humidity) could also be included in the input parameters, together with a full explanation of the raw materials. Moreover, additional in-depth investigations, checks, and effects should be incorporated in order to improve the evaluation and comprehension of the outcomes obtained through the use of ML techniques.

## Figures and Tables

**Table 1:** Descriptive analysis of input variables.

Parameter	Input Variable (kg/m^3^)						
	Fine Aggregate	Coarse Aggregate	Cement	Water	Superplasticizer	Fly Ash	Blast Furnace Slag
Mean	764.4	956.1	265.4	183.1	7.0	62.8	86.3
Standard Error	3.5	4.1	5.1	0.9	0.3	3.2	4.3
Median	769.3	953.2	261.0	185.0	7.8	60.0	94.7
Mode	755.8	932.0	313.0	192.0	0.0	0.0	0.0
Standard Deviation	73.1	83.8	104.7	19.3	5.4	66.2	87.8
Range	398.6	344.0	438.0	125.2	32.2	200.1	359.4
Minimum	594.0	801.0	102.0	121.8	0.0	0.0	0.0
Maximum	992.6	1145.0	540.0	247.0	32.2	200.1	359.4

**Table 2:** Statistical checks of techniques employed.

Model	MAE	RMSE
Support vector regression	3.329	5.325
AdaBoost	2.947	3.908
Random forest	2.223	3.183

**Table 3:** K-fold cross-validation outcomes.

K-Fold	SVR			AdaBoost			Random Forest		
	MAE	RMSE	R^2^	MAE	RMSE	R^2^	MAE	RMSE	R^2^
1	4.37	5.68	0.77	6.79	7.54	0.83	3.94	5.33	0.80
2	7.38	10.25	0.73	6.79	10.10	0.75	5.78	8.11	0.88
3	13.38	19.87	0.80	10.88	14.83	0.89	8.68	12.08	0.66
4	20.13	37.40	0.61	14.46	26.74	0.60	6.65	8.79	0.61
5	9.28	12.69	0.67	7.62	10.93	0.90	6.31	9.62	0.91
6	9.67	12.13	0.75	9.85	11.60	0.77	6.05	7.14	0.84
7	7.91	11.28	0.58	7.60	11.24	0.88	6.79	8.70	0.62
8	4.66	5.95	0.74	4.73	5.21	0.65	7.09	8.59	0.92
9	6.08	9.75	0.55	7.07	8.95	0.61	5.57	5.39	0.79
10	7.51	11.15	0.69	6.06	9.22	0.72	8.25	10.11	0.86

## Data Availability

Not Applicable.
